# Molecular and Biological Mechanisms Underlying Gender Differences in COVID-19 Severity and Mortality

**DOI:** 10.3389/fimmu.2021.659339

**Published:** 2021-05-07

**Authors:** Zena Wehbe, Safaa Hisham Hammoud, Hadi M. Yassine, Manal Fardoun, Ahmed F. El-Yazbi, Ali H. Eid

**Affiliations:** ^1^ Department of Biology, American University of Beirut, Beirut, Lebanon; ^2^ Department of Pharmacology and Therapeutics, Beirut Arab University, Beirut, Lebanon; ^3^ Biomedical Research Center, Qatar University, Doha, Qatar; ^4^ Department of Pharmacology and Toxicology, American University of Beirut, Beirut, Lebanon; ^5^ Department of Pharmacology and Toxicology, Faculty of Pharmacy, Alexandria University, Alexandria, Egypt; ^6^ Department of Basic Medical Sciences, College of Medicine, Qatar University Health, Qatar University, Doha, Qatar; ^7^ Biomedical and Pharmaceutical Research Unit, Qatar University Health, Qatar University, Doha, Qatar

**Keywords:** COVID-19, SARS-CoV-2, vaccine, ACE-2, gender difference

## Abstract

Globally, over two million people have perished due to the recent pandemic caused by SARS-CoV-2. The available epidemiological global data for SARS-CoV-2 portrays a higher rate of severity and mortality in males. Analyzing gender differences in the host mechanisms involved in SARS-CoV-2 infection and progression may offer insight into the more detrimental disease prognosis and clinical outcome in males. Therefore, we outline sexual dimorphisms which exist in particular host factors and elaborate on how they may contribute to the pronounced severity in male COVID-19 patients. This includes disparities detected in comorbidities, the ACE2 receptor, renin-angiotensin system (RAS), signaling molecules involved in SARS-CoV-2 replication, proteases which prime viral S protein, the immune response, and behavioral considerations. Moreover, we discuss sexual disparities associated with other viruses and a possible gender-dependent response to SARS-CoV-2 vaccines. By specifically highlighting these immune-endocrine processes as well as behavioral factors that differentially exist between the genders, we aim to offer a better understanding in the variations of SARS-CoV-2 pathogenicity.

## Introduction

As of March 2021, the World Health Organization (WHO) reported over 100 million confirmed cases of coronavirus disease-2019 (COVID-19) and approximately 2.7 million deaths (1). The available data demonstrates a higher rate of male severity and mortality associated with COVID-19 (2). Even when female infection rates supersede that of males, men commonly present with higher intensive care unit (ICU) admissions and death rates (3). In fact, while females predominated mild and serious cases, there were three times as many male severe pneumonia cases compared to females (4). In addition, the male case fatality rate upon COVID-19 infection, has been consistently greater in countries throughout the world, including England (approximately 27% male: 15% female), Italy (17%: 8%), Spain (10%: 6%) and China (5%:3%) (5). Males generally constituted over 50% of COVID-19 related deaths, as indicated in China (64%), South Korea (53%) and the United Kingdom (UK) (61.7%) (3, 6, 7).

Significant gender-based differences appear to be a common theme in viral infections. Indeed, studies have demonstrated higher incidence and increased fatality rates in males compared to females upon infection with human coronaviruses, SARS-CoV-1 and Middle Eastern Respiratory Syndrome coronavirus (MERS-CoV) (8, 9). However, only a few studies have investigated causes for the discrepancy, which primarily cite an increased pro-inflammatory state in males (10, 11). On the other hand, young females emerged as the most vulnerable group to both the influenza and avian H5N1 virus (12). Moreover, during the 1957 H2N2 pandemic in the United States, the mortality rate of females was higher than males in the same age group of 1-44 years (13). Conversely, hepatitis B virus (HBV) and hepatitis C virus (HCV) are more prevalent in males and this been attributed to the sex hormones (14–16). Some studies linked the relatively higher HBV titer in male serum to testosterone and androgen receptors used to activate HBV genome transcription (16, 17). Testosterone can also increase scavenger receptors required for HCV viral entry (18, 19). By contrast, estrogen downregulates HBV transcription *via* estrogen receptor alpha (ERα), while selective estrogen receptor modulators (SERMs) inhibit the production of mature HCV (19, 20).

These studies highlight that, depending on the virus, an interplay of different factors like sex hormones and inflammatory processes, may allow the symptoms to manifest more severely in one of the genders. Therefore, it is important to characterize unique host factors involved in the pathogenicity of a specific virus, and how these components differ between males and females. Because the reasons for the apparent gender bias in COVID-19 patients are not completely elucidated, we explore the contribution of several relevant host factors that may drive the increased severity of symptoms detected in males. Many of these components include underlying comorbidities, immuno-endocrine processes, signaling molecules, and host proteases, among many others. Understanding how these may render more protection for females would provide more effective measures for managing COVID-19.

## Gender Differences in Comorbidities and the Link to Creatinine

Comorbidities are highly prevalent among COVID-19 fatalities, especially hypertension, diabetes, cardiovascular disease (CVD), and chronic lung diseases (21, 22). Given that the severity and death rates are more pronounced in males, it would be plausible to assume that underlying conditions drive this bias. However, at first glance the relationship between comorbidities, gender and COVID-19 severity is not straightforward. In older individuals, more women (45%) than men (32%) have multiple morbidities (23). Type 2 diabetes (T2D), on the other hand, is equally prevalent in older males and females (24). In addition, deaths due to chronic obstructive pulmonary disease are more prominent in females (25, 26). Moreover, it was demonstrated in a particular study that a higher prevalence of CVDs is associated with postmenopausal women and that females are more prone to ischemic heart disease (27). To further corroborate the obscure link between comorbidities, gender, and COVID-19 severity, it was shown that there was no gender difference in the prevalence of comorbidities in those who perished, including the most common diseases of hypertension, diabetes, CVDs, and chronic lung diseases (4).

Although prevalence of comorbidities was common between the two genders, one of the most striking differences that did exist was the exceptionally elevated levels of creatinine in blood samples of males compared to females (4). High creatinine levels were detected in individuals with severe cases of COVID-19 who were transitioning to multi-organ failure (28). Elevated creatinine serum levels are often corollary to comorbid diseases including diabetes, cancer, chronic renal failure, and some viruses, which can eventually lead to acute kidney injury (AKI) in severely ill and hospitalized patients, including COVID-19 patients (29, 30). The development of AKI in patients with comorbidities is positively correlated with increased mortality. Up to 7% of hospitalized patients and 20%-50% of ICU patients develop AKI, of which 5% require renal replacement therapy (29). Stimulation of the inflammatory response, either to an underlying disease or infection, results in abundant expression of cytokines, like tumor necrosis factor alpha (TNFα) and interleukin 6 (IL6), which lead to endothelial activation of adhesion molecules and further chemokine production (28). Ultimately, leukocytes from the blood infiltrate the interstitial tissue of the kidney, disrupting its processes. The cytokine storm that arises in COVID-19 patients can further exacerbate this state (28). Importantly, it has been shown, that for the same disease, women develop lower levels of serum creatinine than males, like in the case of chronic kidney disease (31). Because comorbid diseases are shown to be equally prevalent in those who have perished from COVID-19, their physiological response- as demonstrated by a highly significant creatinine increase in males compared to females- may be one of the differential contributors to increased disease severity in men (4).

## Gender Differences in Cardiac Repair Mechanisms

Notably, patients with underlying cardiovascular related pathologies such as hypertension and coronary heart disease (CHD) develop more detrimental COVID-19 symptoms (32–34). Additionally, CVD is one of the highest risk factors associated with COVID-19 severity and mortality (33–35). Therefore, it may be expected that severely infected males would have a higher CVD prevalence and severity. However, like other morbidities, CVD epidemiological data does not fully support this assumption. For example, while age-adjusted men have a higher incidence of CVD, it is the leading cause of mortality and hospitalizations in females (36). In fact, after the age of 50, women show higher CVD events (37). On the other hand, hypertension is more likely to lead to heart failure in males, rather than females (38). Given the more pronounced CVD symptoms in females and that it is one of the most common underlying co-morbidities in severe COVID-19 patients, it somewhat counter-intuitive that males would predominate the deaths resulting from SARS-CoV-2 (33). An alternative perspective would involve examining gender differences in cardiac repair mechanisms, following SARS-CoV-2 induced cardiac injury.

Direct cardiac injury due to the interaction between SARS-CoV-2 and cardiomyocytes, in addition to the concomitant systemic inflammation, is the primary mechanism by which the virus induces myocardial injury (39). Therefore, it is important to highlight sexual dimorphism which also may exist in cardiac restoration post-injury. Indeed, it was shown that the repair response after injury differs between males and females (38). In animal models, females displayed improved survival in the event of acute heart failure (38). This is associated with limited cardiac remodeling and more efficient functional recovery. Uniquely, females produced amplified levels of reparative leukocytes and epoxy-eicosatrienoic acids, which exert anti-hypertensive and anti-inflammatory effects on blood vessels (38). Collectively, such a female response is associated with a more adequate regulation of inflammation and survival post-cardiac injury. As such, although older females tend to have a higher incidence of CVD compared to age-matched males, it is possible that the reduced female mortality related to COVID-19, may partially be attributed to an enhanced reparative response following cardiovascular injury.

## Gender Differences in Host Molecular Components Used by SARS-CoV-2

### Cell Receptors

#### Angiotensin I-Converting Enzyme 2 (ACE2) Expression

One potential contributor to the apparent gender disparity in COVID-19 may be ACE2 expression. This enzyme has been established as the route of entry for SARS-CoV-1 and SARS-CoV-2, upon binding of its spike (S) protein, and it is abundantly expressed in the lungs and heart (40). SARS-CoVs reduce bioavailable ACE2 levels by competitive inhibition and by subsequent cleavage of the active site ectodomain (41–43). The loss of ACE2 is associated with increased lung edema, reduced lung function and diminished protection against acute lung injury and, sometimes, severe acute lung failure (42, 43). In addition, it has been suggested that higher levels of ACE2, ultimately lead to an increase in soluble ACE2 ectodomains in circulation (44). Subsequently, these enzymes would serve as circulating scavengers for SARS-CoV-2, thus limiting their interaction with cell-bound ACE2.

The *ace2* gene locus lies on the X chromosome (45). Some scientists suggested that the presence of two alleles from the two X chromosomes in females, compared to males with only one X chromosome, may exert a protective effect (45). However, given that one of the X chromosomes is permanently inactivated in females, it is not likely that heterozygous alleles for ACE2 would emerge in a female. In addition, polymorphism analysis of ACE2 indicated that there are no significant sex-related differences (46). Therefore, while it may be an obvious contender for gender disparities in COVID-19, the effect is likely not associated with sex-related single nucleotide polymorphisms (SNPs).

ACE2 expression in rat lungs is significantly diminished in both genders upon aging (47). However, the reduction is more pronounced in older male rats, which display a 78% reduction in ACE2 expression, in contrast to older females which demonstrated a 67% decrease (47). This has been partially linked to the reduction of sex hormones in aging females and males (48). ACE2 is also reduced in individuals with chronic diseases (48).

According to this data, the individual with the lowest ACE2 expression would be an older male with comorbidities. The added burden of a COVID-19 infection would further jeopardize their already depressed ACE2 levels. This may partially explain their relatively higher severity (as reflected by ICU admissions) and mortality due to COVID-19 (33). However, it is important to also evaluate the change, if any, in ACE2 expression upon SARS-CoV-2 infection, and if the response differs between genders.

#### Neuropilin-1 (NRP1)

The S protein in SARS-CoV-2 contains a furin cleavage site which is absent in the S protein of other coronaviruses (49). This added site may contribute to enhanced viral binding and host cell invasion upon its cleavage (50). While ACE2 binds to the receptor binding domain (RBD) on the S protein, another cell receptor, neuropilin-1 (NRP1), has recently been shown to bind to the furin-cleavage site on the S1 segment of the S protein (51). Incidentally, endothelial and respiratory epithelial cells express the highest level of NRP1 in the body, which may explain the excessive damage exerted upon lung and myocardial tissue particularly in COVID-19 patients (50). Indeed, blocking NRP1 with monoclonal antibodies greatly reduced SARS-CoV-2 infection of cells in *in vitro* conditions (51).

NRP1 is often studied in the context of tumorigenesis as it is a receptor for vascular endothelial growth factor (VEGF) and contributes to the cancer hallmarks of angiogenesis, invasion and metastasis (52). In fact, it is often upregulated in several cancers, including stomach adenocarcinoma, squamous cell carcinoma and melanoma (52–55). Although NRP1 expression increases according to the size of these tumors and degree of metastasis, there is no significant difference in expression between males and females (55). Studies comparing baseline NRP1 tissue expression of men and women without cancer or those with various diseases, however, are still lacking. Elucidating this component would establish whether or not NRP1 partially drives the gender disparity associated with COVID-19 severity.

#### CD147

Cluster of differentiation 147 (CD147), also known as basic immunoglobulin (basigin) or extracellular matrix metalloproteinase inducer (EMMPRIN) is a membrane receptor shown to interact with SARS-CoV-2 (56). In fact, inhibition of this receptor by meplazumab was shown to reduce SARS-CoV-2 amplification (56). It is present in several tissues in the body, and it is especially overexpressed in inflamed tissues. As its name suggests, it induces extracellular matrix proteases, which may contribute to the development of fibrosis. Incidentally, it is relatively abundant in fibrotic lung tissue compared to normal lung tissue (57).

A CD147 expression study has demonstrated that it is not differentially expressed between males and females in primary bronchial epithelial cells or in blood cells (58). However, gender differences were detected in gastric cancer cells, in which males had significantly higher expression of CD147 and this was correlated to a worse prognosis (59). It has yet to be examined whether or not there is differential CD147 expression between men and women at homeostatic conditions or in the presence of other underlying diseases.

### Renin-Angiotensin System

The renin-angiotensin system (RAS) has been characterized as the main homeostatic regulator of extracellular fluid *via* renal, cardiovascular, and cerebral interactions (60). Alterations in fluid balance trigger a series of enzymatic cleavages beginning with renin and culminating in the production of angiotensin II (AngII) by angiotensin-converting enzyme 1 (ACE1) (61). Upon binding with high affinity to angiotensin receptor 1 (AT1), AngII initiates a variety of responses that include vasoconstriction and renal sodium retention (61). On the other hand, the metabolism of AngII to angiotensin 1-7 (Ang1-7) by ACE2 can counteract these activities (62, 63). After identifying RAS components on a variety of other tissues, other roles became evident (62). For example, the ACE1/AngII arm also results in pro-inflammatory, pro-fibrotic and pro-oxidative effects (64–66), while the ACE2/Ang1-7 arm counteracts these processes (64, 67). As such, the ACE2/Ang1-7 axis is mainly characterized as the protective arm of RAS.

Studies have indicated that females have significantly higher blood levels of Ang1-7 compared to males, but similar levels of AngII (68, 69). Correspondingly, these women also had a lower incidence of hypertension compared to males with the same body mass index (BMI) and waist circumference (68, 69). The higher levels of Ang1-7 may enable females to counteract inflammation, such as that induced by COVID-19, more effectively. In fact, supplementation with Ang1-7 is currently being suggested as treatment against SARS-CoV-2 (70).

### Signaling Molecules

#### ERK

Mitogen activated protein kinase (MAPK) pathways involve the sequential phosphorylation of three MAP kinases and culminate in the activation of a downstream effector involved in cellular growth, proliferation, differential, or death (71). An extracellular signal-regulated kinase (ERK) is a major MAPK and is significantly upregulated in most comorbidities present in COVID-19 patients like ischemic heart, CVD, hypertension, diabetes, and cancer (71–77).

It has been shown that several viruses, including some coronaviruses like SARS-CoV-1, Middle East respiratory syndrome (MERS) and likely SARS-CoV-2 also induce ERK for viral replication, and its inhibition results in depressed viral progeny (78, 79). Moreover, cells infected with SARS-CoV-1 display upregulation of ERK and its downstream pro-inflammatory cytokine and monocyte chemoattractant protein-1 (MCP-1) (80). Other cytokines are likely to be potentiated *via* the same mediator, thus contributing to the detrimental cytokine storm induced by SARS-CoV-2 (80). As such, enhanced ERK activation in chronic inflammatory diseases may amplify SARS-CoV-2 replication and pathogenicity.

Animal studies have demonstrated that ERK is differentially activated in males and females during inflammation (81). Specifically, male- and female-derived rat aortic smooth muscle cells (RASMCs) were induced with conditions of abdominal aortic aneurysm (AAA) (81). As a result, activated (phosphorylated) ERK (p-ERK) expression was significantly higher in male derived cells compared to female cells. Moreover, the downstream target, matrix metalloprotease 2 (MMP-2), which is involved in tissue remodeling, was also correspondingly increased in males (81).

A relatively higher expression of ERK in males compared to females has also been corroborated in human neutrophils, particularly in the context of leukotriene (LT) synthesis by 5-lipoxygenase (5-LO) (82). Production of LTs is preceded by 5-LO translocation from the cytoplasm to the nuclear envelope. Trafficking is more pronounced in activated female neutrophils and highly lacking in males. Translocation of 5-LO is not required in male neutrophils because it already resides in the peri-nuclear region, consequently limiting LT production (82). This phenomenon is due higher levels of dihydrotestosterone (5α-DHT), which activate ERK, thus maintaining a constant localization of 5-LO near the nuclear envelope. These findings indicate that male sex hormones can rapidly induce and sustain enhanced expression of ERK, possibly *via* 5α-DHT receptors like those that may exist on neutrophils (82).

These studies demonstrated in both inflammatory states (AAA) and normal physiological states (neutrophils) that ERK activation is relatively higher in males. The apparent male bias in COVID-19 severity could partially be attributed to higher basal levels of ERK expression, which are further exacerbated in chronic diseases, thus enhancing viral replication and associated tissue damage.

#### STAT1

One of the most rapid anti-viral responses in the host involves the secretion of type I interferons α and β (IFNα/β) (83). Type I IFN receptor is present throughout most host cells and activates the downstream JAK1/STAT1 (Janus kinase 1/signal transducer and activator of transcription 1) signaling pathway (83). STAT1 predominantly induces the expression of genes that hinder viral replication (83). Incidentally, coronaviruses like SARS-CoV-1 are sensitive to the activity of type I IFNs, and their growth can be hindered by the addition of IFNα/β. However, SARS-CoVs do not endogenously induce IFNα/β in infected host cells, which contributes to their severe and prolonged pathogenicity due to their ability to evade the initial immune response (83).

In animal models, healthy females have presented with higher baseline expression of IFNα-induced genes in immune cells, such as B lymphocytes (84). This phenomenon was linked to 17-β-estradiol in females, which further augments JAK1/STAT1 signaling (84). Moreover, estrogen signaling *via* ERα has been shown to promote type I IFN synthesis (85).

Moreover, in certain chronic diseases, STAT1 in females is more upregulated compared to age-matched males (84). These findings suggest that higher STAT1 levels in females, especially in immune cells, may equip them with a more ‘prepared’ initial anti-viral response. It may also offer insight into why females, who have a higher prevalence of the underlying chronic diseases, present with less COVID-19 severity and mortality compared to males.

### Host Proteases Associated With SARS-Cov-2 Entry

#### ADAM17

Efficient cellular entry of SARS-CoVs involves adhesion of the outer spike protein (S) to the membrane bound ACE2 (86, 87). Thereafter, the membrane-bound enzyme, a disintegrin and metalloproteinase 17 (ADAM17), cleaves the active site ectodomain of ACE2, shedding it into the plasma (88). Inhibition of ADAM17 is associated with lower coronavirus entry into a cell, indicating its relevant, yet covert, role in viral uptake (89). ADAM17 also cleaves and activates a number of immunomodulatory membrane-associated proteins like tumor necrosis factor α (TNFα) (90, 91). In fact, concomitant to ACE2 cleavage, is increased activation of TNFα, which is thought to initiate the tissue damage associated with viral invasion (89).

Gender differences in ADAM17 activity and expression are generally lacking. However, sex disparities in ADAM17 have been detected in relation to AAA (92). A particular SNP in the ADAM17 gene was linked to a higher expression and activity of ADAM17, and an increased risk of AAA in males compared to females (92). It may be worth examining if pulmonary ADAM17 activity varies between males and females to better understand the gender gap in SARS-CoV-2 severity.

#### TMPRSS2

Indispensable for SARS-CoV-2 entry into host cells is the transmembrane serine protease 2 (TMPRSS2) (87). Upon cleaving the S protein at S1 and S2’, TMPRSS2 facilitates the initial partitioning of this protein into the host cell membrane (93). TMPRSS2 has previously been established as a gender biased protein as it is regulated by an androgen-sensitive promoter and is most abundantly expressed in prostate cells (94). Four androgen receptor (AR) binding sites (ARBs) are located in the promoter region of *TMPRSS2*, two of which respond to androgen stimulation and require the transcription factors GATA Zinc Finger Domain-Containing Protein 2A and 2B (GATA2A and GATA2B, respectively) for efficient transcription (94).

In animal studies, it was previously shown that GATA transcription factors, including GATA2, are differentially expressed in male and female tissues, particularly at different stages of development (95). It is not yet known if SARS-CoV-2 enhances GATA expression and if there is gender disparity in its expression. Moreover, because *TMPRSS2* is androgen-responsive, it would therefore be tempting to expect TMPRSS2 expression to be elevated in males. However, a study examining lung TMPRSS2 expression in the lungs indicated that there is no significant difference between males and females regardless of the age group (46). Nevertheless, this does not necessarily rule out differential expression in other tissues.

Although expression levels did not differ, several distinct SNPs are prevalent in the *TMPRSS2* gene. In fact, one of the most prominent variant haplotypes, termed the “European” haplotype was completely absent in Asians (46). Importantly, this haplotype is suggested to have an enhanced androgen-response for TMPRSS2 expression (46). It is not yet known if this haplotype is also more prevalent in males. Because TMPRSS2 plays an integral role in viral invasion, a thorough examination of its expression in various tissues among males and females – in both homeostatic and pathological conditions- would further clarify the male bias in COVID-19.

#### Furin

Another important membrane protease associated with SARS-CoV-2 viral entry is furin (50). The furin cleavage site (FCS) is located upstream of the S1 cleavage site in the S protein. The FCS is not present in SARS-CoV-1 and it is thought to lend a greater capacity for SARS-CoV-2 to infect cells- however this is still disputed (49). It has been demonstrated, though, that deletion of the FCS within the S protein is associated with reduced replication in respiratory cells and pathogenesis *in vivo* (50). Gender disparity in furin expression has been previously demonstrated in asthmatic patients. Specifically, bronchial cell samples obtained from asthmatic patients revealed that furin is positively associated with the male gender and this was speculated as a possible factor contributing to COVID-19 severity (96). Further analysis of genetic variations at the FCS and any associated gender differences may offer insight to the more pronounced COVID-19 severity and mortality in males.

## Gender Differences in the Immune Response

### Immune Response Background

An effective immune response is essential for viral elimination, yet, upon over-activation, as in some chronic conditions, it can induce cellular injury and lead to aberrant antigenic response with immunological pathology (97). Activation of host cell pattern recognition receptors (PRRs) in response to viral pathogen-associated molecular patterns (PAMPs) in innate immune cells, including dendritic cells and macrophages, initiates the response of the innate immune system. Specifically, PRRs which include toll-like receptors (TLRs) and retinoic acid-inducible gene I-like receptors (RLRs), lead to downstream activation of the JAK/STAT pathway, resulting in the production and secretion of pro-inflammatory cytokines particularly type I and III interferons (IFNs) (98, 99). Other pro-inflammatory molecules secreted include interleukin-12 (IL-12), TNFα, and chemokine ligand 2 (CCL2), and upon uncontrolled expression, are collectively responsible for the associated pulmonary injury (98, 100). As a consequence, the degree to which the virus activates PRR influences the magnitude of immune cell recruitment and inflammatory cytokine production.

### Gender Disparities in the Innate and Adaptive Anti-Viral Immune Response

Sexual dimorphism in the susceptibility and response to viral pathogens has been previously documented in both clinical and pre-clinical studies. In general, females seem to be less susceptible to viral infections. Indeed, females mount higher innate immune responses than males, resulting in a faster viral recognition and production of IFN and inflammatory cytokines, inducing more rapid viral clearance (101, 102). In fact, the activity and number of innate active immune cells, including macrophages, monocytes, and dendritic cells (DCs), are higher in females than males (103–105). In addition, the innate activity of PRRs in detecting viral nucleic acids is more effective in females (104). For example, during viral infection, TLR7 – a PRR that detects single stranded viral RNA- stimulates higher production of IFNβ in females (97). These findings suggest that upon infection with SARS-CoV-2, females may be better equipped to initially respond, and attenuate viral invasion and pathogenicity compared to males.

**Figure 1:**
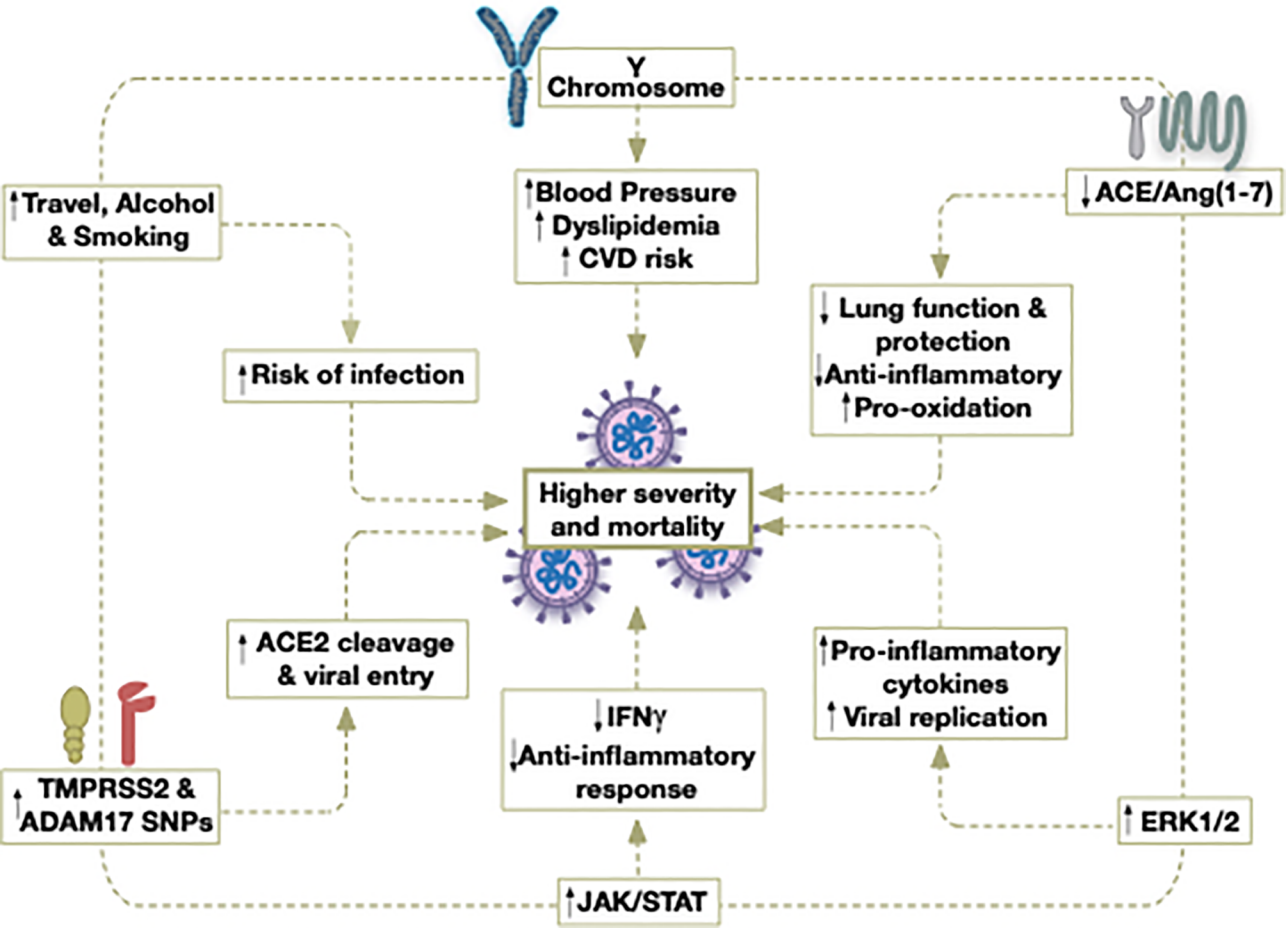
Potential contributors to the higher severity and mortality caused by COVID-19 in older males.

Gender differences have also been detected in the adaptive immune response, where females exhibit a higher humoral and cell-mediated immune responses than males (106). In females, antigen-presenting cells (APC) are more efficient at presenting peptides than males (107). Thus, communication between the innate and adaptive cells may occur more rapidly in females once SARS-CoV-2 is detected. In fact, following the activation of the adaptive immune cells by APC, the levels of antibodies (106, 108), as well as immunoglobulins, are greater in females than males in both human and experimental murine models (109, 110). Specifically, upon viral infection, females exhibit higher levels of cluster of differentiation 4 (CD4^+^) T cells, thus activating a greater number of effector T cells (111, 112). Additionally, a more robust inflammatory/cytotoxic T-cell response along with an upregulation of the pro-inflammatory genes is reported in females, indicating that viral clearance is also more adequate (113) (**Figure 1**).

Based on this, one can deduce that females, and due to their stronger, more effective anti-viral immunity beginning from initial viral invasion and culminating with viral clearance, are less prone to develop severe viral infections than males do. These findings may also be extended to the response of females after infection with SARS-CoV-2. However, it should be noted that, following viral clearance and return to homeostasis, a prolonged immune system activation in women may contribute to increased development of immunopathology associated with viral infections (101, 108). On the other hand, the lower immune response to the viral infection at homeostasis is responsible for the persistent viral infections seen in males (101, 108). The seemingly more robust immune response present in females may be partially attributed to the role of sex hormones.

As for SARS-CoV-2, what is known till now is that once the virus is recognized by alveolar macrophages, it will trigger the generation of pro-inflammatory cytokines and chemokines (114–116). Studies have shown that patients with severe symptoms, a dysfunctional immune response occurs caused by hyperactivation of macrophages and monocytes, which triggers a cytokine storm mediating a widespread inflammation and thus multi-organ damage (114, 116). Cytotoxic T cells are thereafter recruited and Th1/Th7 cells stimulation could lead to aggravated inflammatory response (117). B cells recognize viral proteins and are activated to produce SARS-CoV-2 specific antibodies (118). The detected humoral response directed against SARS-CoV-2 has been found to be of great similarity with other coronavirus infections, initiating the production of specific immunoglobulin G and M (IgG and IgM) (119, 120). These specific antibodies produced aid in viral deactivation as well as providing systemic immunity in different organs (117). Though studies are now compiling, it seems that SARS-CoV-2 possess similar cellular invasion mechanisms as SARS-CoV-1 and as such a similar trend in gender differences could be observed.

### Estrogen Commonly Enhances the Innate and Adaptive Immune Response

Apart from factors merely related to discrepancies in the immune and inflammatory responses, other factors, including sex hormones, act as key modulators of immune responses (121). Receptors for sex hormones have been identified on immune cells, suggesting a direct effect of those hormones on the immune response (122). The binding of hormones to their respective receptors directly alters cell signaling pathways, including nuclear factor kappa-light-chain-enhancer of activated B cells (NF-kB), the transcription factor cJun, and IFN regulatory factor (IRF), resulting in variable production of cytokines and chemokines, in addition to regulating the development and maturation of immune cells (122).

**Figure 2:**
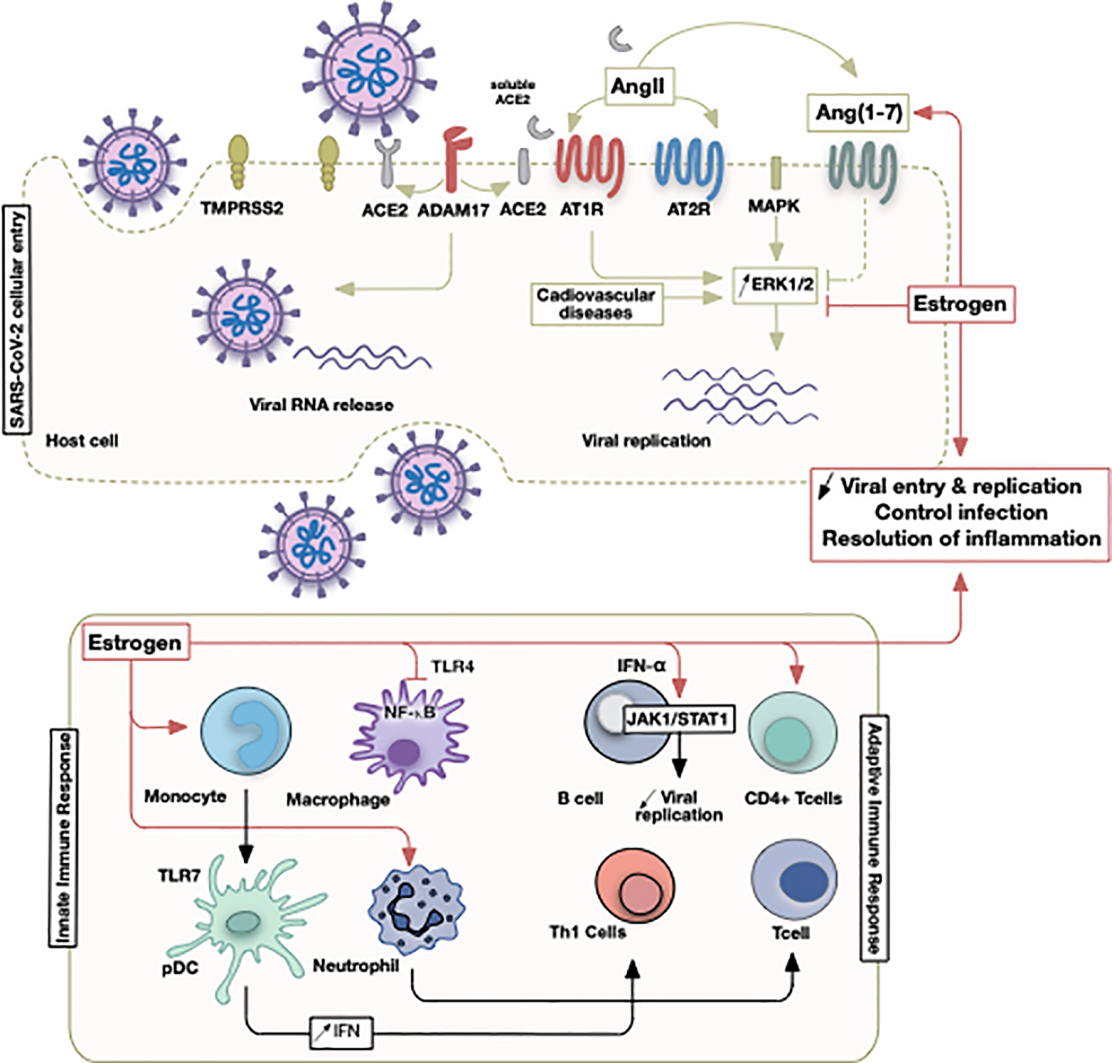
Illustrative summary of events involving estrogen in COVID-19. The initial SARS-CoV-2 viral entry and recruitment of innate and adaptive immune cells culminate in a pathological state following viral infection or successful control of infection. Sex discrepancies could be pertinent due to estrogen hormone that operates at different points as indicated.

Estrogen receptor (ER) subtypes ERα and ERβ, are expressed in various immune cells such as lymphocytes, macrophages, and dendritic cells (85). ERα appears to be the key regulator in cellular differentiation of hematopoietic progenitor cells (123). ERs induce activation of surface receptors (as insulin growth factor), ERK/MAPK, protein kinase C (PKC) and cyclic adenosine monophosphate (cAMP) signaling (124, 125). Additionally, ERs expressed on leukocytes modulate their function: low levels of estrogen tend to increase the synthesis of pro-inflammatory cytokines and high levels decrease their production (**Figure 2**) (108, 126). Moreover, estrogen promotes adaptive T cell response against viral infection through increasing neutrophil accumulation (127). It also induces monocyte differentiation into inflammatory DC, inducing greater production of cytokines and IFNs and activates T helper 1 cells (Th1) (108). Additionally, estrogen inhibits TLR4-mediated NFκB activation in macrophages *via* suppression of micro-RNAs and increases anti-inflammatory type-1 IFN *via* TLR (10, 11). Estrogen also regulates STAT activity by increasing the expression of cytokine inhibitor proteins in T cells and macrophages (128). ER activation suppresses the expression of CC-motif ligand-2 (CCL2) in leukocytes, therefore decreasing their migration (128). Estrogen has a crucial role in modulating zinc finger proteins that regulate various metabolic functions, cell cycle, and lipid metabolism pathways involved in the progression of influenza A infection (129).

While estrogen often activates target immune cells, testosterone and its active metabolite suppress immune cell activity as well as the production of inflammatory cytokines (130, 131). Specifically, stimulation of androgen receptors reduces the production of pro-inflammatory cytokines (132). Indeed, androgen deficiency in males is associated with high levels of inflammatory cytokines and increased CD4+/CD8+ T-cells ratio (132, 133).

Despite these findings, it is essential to highlight that age-related changes to innate immunity should not be overseen, which have previously been described (134). Primarily, levels of hormones in both males and females are not always stable. Estrogen levels in females vary during the menstrual cycle, pregnancy and fall after menopause; however, in males, testosterone levels remain stable until 60 years of age. More importantly, high estrogen levels decrease innate immunity, increase Th2-induced responses, and enhance humoral immune activity (135). Therefore, women, based on their age and estrogen and receptor levels, might present multiple reactions to viral infections. Nevertheless, these overall findings suggest that the commonly pronounced levels of estrogen in females are driving a more robust response against the infection with SARS-CoV-2.

### Selective Estrogen Receptor Modulators as a Treatment Against COVID-19?

As presented earlier, over expression of ERs was found to suppress viral replication (136). Repurposing selective estrogen receptor modulators (SERMs) as anti-viral drugs had led to their use against Ebola virus, human immunodeficiency virus (HIV), and HCV infections (137). SERMs were found to interfere at the post-viral entry step and suppress Th1 cells while inducing Th2 cytokine expression (138). A particular SERM, tamoxifen, has been repurposed for anti-viral activity in cases of vesicular stomatitis as well as HCV infections. It inhibits vesicular stomatitis viral replication by enhancing type I IFN response and macrophage stimulation (139). Moreover, Tamoxifen targets several steps in HCV infection *via* ERα signaling (20). HCV replication, post replication, viral attachment on the cell surface as well as viral entry were inhibited by Tamoxifen (20).

Another SERM drug, Raloxifene, was found to reduce influenza A virus titer in human female nasal epithelial cells (129). Viral infections by MERS-CoV and SARS-CoV in established cell lines were inhibited by Toremifene, a nonsteroidal SERM (140). The drug’s mechanism of action involves interaction with CoV membrane glycoproteins, thereafter destabilizing it and inhibiting its replication (140).

Importantly, ERs are expressed in the upper and lower respiratory tract and targeting these receptors in respiratory viral infections is of great importance (141). The presence of ER implies an important role of estrogen in pulmonary cellular physiology and offers a possible route for protecting against lung diseases using SERMs. Importantly, many women currently taking SERMs as estrogen replacement therapy or as a treatment for other diseases may benefit from their mitigating effects on spontaneous viral infections (127). Altogether, SERMs (like Toremifene and Raloxifene) could offer a possible add on treatment for COVID-19.

## Behavioral and Social Gender Differences

Gender discrepancies in the exposure to viral infections are due to evident disparities in social roles, activities, and behaviors (142). Proper assessment of differences in disease exposure is warranted when studying gender discrepancies in the incidence of infections. Males tend to have more social contacts, travel more frequently, and spend more time in settings that could be conducive to viral transmission (143). In addition, gender roles influence how and where males and females spend their time and the quality of healthcare they receive. Moreover, gender differences in access to healthcare facilities in specific countries may lead to further variability in disease progression. For example, boys in India usually have better follow up from healthcare professionals and medical care than girls (144).

Additionally, behavioral factors are involved in gender discrepancies affecting the burden of viral infections (145–147). Alcohol consumption and cigarette smoking exert complex and detrimental effects on systemic and pulmonary immunity. Both factors suppress a wide range of immune responses, therefore, predispose individuals to the acquisition of further infections (145, 146, 148). Alcohol drinking and cigarette smoking habit are higher in males than females, which would therefore render men more prone to alterations in the immune responses (145, 146, 149–151). For example, the risk of tuberculosis infection is seen to be higher in men than in women (147). Furthermore, high alcohol consumption is linked to liver cirrhosis as well as increased mortality and morbidity rates. Recent studies have documented the association of liver diseases as well as smoking with a higher risk for severe COVID-19 and mortality; therefore, smoking and alcohol cessation should be encouraged (152, 153).

## Gender Differences in Vaccine Response

COVID-19 vaccine development is expected to be the most effective way to control the pandemic and combat this novel virus. It is quite interesting that besides the role of sexual differences in infection immunity, sexual differences are documented to be important in the immune response to vaccines (154, 155). Females do mount greater immune response to vaccinations than men thus facilitating vaccine efficacy, yet they more commonly experience severe side effects (154, 156, 157). Unfortunately, there is serious lack of attention to sexual dimorphism in vaccine clinical trials.

There are currently several vaccines against SARS-CoV-2 that have been rolled out (158). Early reports of clinical trials for SARS-CoV-2 vaccination have not addressed sexual differences (159–161). Studies reported promising immunogenicity and safety outcomes. In addition, local and general adverse effects are being addressed yet not segregated by gender (160, 161). However, a clinical trial of the adenovirus-vector vaccine candidate measured adverse effects outcomes and reported that females experienced side effects such as fever more commonly than males (162).

## Concluding Remarks

To date, COVID-19 still represents a worldwide health concern. Culminating evidence demonstrates more favorable COVID-19 outcomes in adult females, especially in the mortality rate. Among the possible factors discussed are those related to females’ molecular factors associated with comorbidities, factors affecting SARS-CoV-2 cellular entry, RAS, specific signaling pathways affecting viral replication, and tissue damage as well as those related to immune-endocrine processes. The data presented above provide potential mechanisms, including the role of sex hormones in the different susceptibility and outcomes of COVID-19 infection (**Table 1**). However, because virus severity is not always lower in females (as demonstrated by some influenza viruses), it is important to recognize unique host cell components exploited by individual viruses and to characterize any associated sexual dimorphism. In our opinion, further epidemiological and molecular studies are needed to identify the key-players in gender bias in response to illness, disease outcomes and more importantly in response to anti-viral treatment modalities.

**Table 1:** Factors contributing to increased COVID-19 severity and mortality in males.

Factor	Higher in Females	Higher in Males	The Effect	Reference
Creatinine		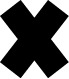	Patients who are males with chronic diseases display highest levels of creatinine.	(29, 30)
			It may lead to acute kidney injury (AKI) in COVID-19 hospitalized patients, increasing risk of death.	
			AKI stimulates release of pro-inflammatory cytokines (TNFα, IL-6), further exacerbating kidney disease.	
			Cytokine storm in COVID-19 patients may exacerbate this process.	(28)
Cardiac Repair Mechanisms	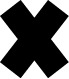		Females have more efficient functional recovery post-cardiac injury.	(38)
			Females produce more reparative leukocytes and anti-hypertensive epoxy-eicosatrienoic acids.	
			SARS-CoV-2 associated cardiac injury may be less pronounced in females due to more efficient cardiac repair.	
ACE2 Expression	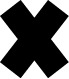		ACE2 is retained in females more than males, even upon aging and the present of chronic disease.	(47, 48)
			Higher levels of ACE2 are more protective against acute lung injury & severe lung failure.	(42, 43)
			Upon ACE2 cleavage, the ectodomains may serve as circulating scavengers for SARS-CoV-2, potentially reducing the viral load.	(44)
NRP1		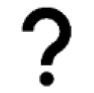	NRP1 binds to the furin-cleavage site on the S1 segment of the S protein. This may increase its cell invasion and pathogenicity.	(51)
CD147		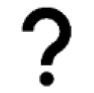	It is a membrane receptor shown to interact with SARS-CoV-2. It induces extracellular matrix proteases, which may contribute to the development of fibrosis. It is abundant fibrotic lung tissue compared to normal lung tissue.	(56, 57)
Ang-1-7	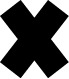		Ang1-7 exerts anti-oxidative and anti-inflammatory activities.	(69)
			Supplementation with Ang1-7 has been suggested as a possible treatment for COVID-19.	(70)
ERK		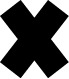	In both homeostatic and inflammatory conditions, ERK is relatively higher in males. Testosterone rapidly induces ERK expression.	(71, 81, 82)
			SARS-CoV-2 replication involves ERK, which may support increased viral progeny in males.	(79)
STAT1	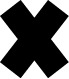		STAT1 inhibits viral replication. SARS-CoVs can evade activation of STAT1. It is relatively higher in females with chronic diseases and in homeostatic conditions.	(83)
ADAM17		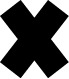	Facilitates viral entry into the host cell by cleavage of S protein. It also cleaves and activates pro-inflammatory cytokines associated with viral-related tissue damage.	(87–89)
TMPRSS2		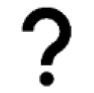	Cleavage of S protein by TMPRSS2 allows viral entry into host cell. It is regulated by an androgen-sensitive promoter and it is greatly expressed in prostate cells. In lungs, TMPRSS2 is not differentially expressed between the genders. Is there gender dimorphism in its expression in other body tissues?	(94)
Furin		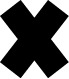	Higher in asthmatic males. Furin cleavage site is located in the S protein and it is required for adequate viral replication and pathogenicity.	(49)
Anti-viral immune response	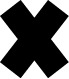		Pattern recognition receptors for detecting viral nucleic acids, antigen presentation of peptides, immunoglobulins, secretion of IL-12, CD4+ T cells and cytotoxic T cells are all more numerous and efficient in females. Innate immune cells (macrophages, monocytes, and dendritic cells) are more numerous in females.	(104)
Estrogen & the immune response	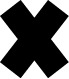		Greater estrogen levels attenuate secretion of pro-inflammatory cytokines, which is mediated via estrogen receptors found on macrophages and other lymphocytes. Estrogen enhances neutrophil accumulation and monocyte differentiation into dendritic cells.	(85, 108, 126)
Behavioral factors		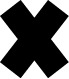	In general, males travel, smoke and consume alcohol more frequently than females, which contribute to more social contact and pronounced detrimental effects on systemic and pulmonary immunity, respectively.	(145, 146, 148)

“X” indicates that this factor is higher in the gender. “?” indicates that it is not known in which gender it is higher.

## Author Contributions

AE conceived of the idea. ZW, SH, MF, and HY wrote the first draft. HY critically edited the first draft. AE-Y and AE did the critical edit of the next four drafts. AE made the final edits on the last draft. All authors contributed to the article and approved the submitted version.

## Conflict of Interest

The authors declare that the research was conducted in the absence of any commercial or financial relationships that could be construed as a potential conflict of interest.

## References

[1] World Health Organization. (2021). Available at: https://www.who.int/emergencies/diseases/novel-coronavirus-2019.

[2] Abduljalil JM Abduljalil BM. Epidemiology, Genome, and Clinical Features of the Pandemic SARS-CoV-2: A Recent View. New Microbes New Infect (2020) 35:100672. 10.1016/j.nmni.2020.100672 32322400 PMC7171182

[3] Shim E Tariq A Choi W Lee Y Chowell G. Transmission Potential and Severity of COVID-19 in South Korea. Int J Infect Dis (2020) 93:339–44. 10.1016/j.ijid.2020.03.031 PMC7118661 32198088

[4] Jin JM Bai P He W Wu F Liu XF Han DM. Gender Differences in Patients With COVID-19: Focus on Severity and Mortality. Front Public Health (2020) 8:152. 10.3389/fpubh.2020.00152 32411652 PMC7201103

[5] Scully EP Haverfield J Ursin RL Tannenbaum C Klein SL. Considering How Biological Sex Impacts Immune Responses and COVID-19 Outcomes. Nat Rev Immunol (2020) 20(7):442–7. 10.1038/s41577-020-0348-8 PMC7288618 32528136

[6] Wu Z McGoogan JM. Characteristics of and Important Lessons From the Coronavirus Disease 2019 (Covid-19) Outbreak in China: Summary of a Report of 72314 Cases From the Chinese Center for Disease Control and Prevention. Jama (2020) 323:1239–42. 10.1001/jama.2020.2648 32091533

[7] Mohamed MO Gale CP Kontopantelis E Doran T de Belder M Asaria M. Sex Differences in Mortality Rates and Underlying Conditions for COVID-19 Deaths in England and Wales. Mayo Clin Proc (2020) 95(10):2110–24. 10.1016/j.mayocp.2020.07.009 PMC7377724 33012342

[8] Karlberg J Chong DS Lai WY. Do Men Have a Higher Case Fatality Rate of Severe Acute Respiratory Syndrome Than Women do? Am J Epidemiol (2004) 159(3):229–31. 10.1093/aje/kwh056 PMC7110237 14742282

[9] Leong HN Earnest A Lim HH Chin CF Tan C Puhaindran ME. SARS in Singapore–predictors of Disease Severity. Ann Acad Med Singapore (2006) 35(5):326–31. 16829999

[10] Murphy AJ Guyre PM Pioli PA. Estradiol Suppresses NF-Kappa B Activation Through Coordinated Regulation of let-7a and miR-125b in Primary Human Macrophages. J Immunol (2010) 184(9):5029–37. 10.4049/jimmunol.0903463 PMC2882792 20351193

[11] Channappanavar R Fett C Mack M Ten Eyck PP Meyerholz DK Perlman S. Sex-Based Differences in Susceptibility to Severe Acute Respiratory Syndrome Coronavirus Infection. J Immunol (2017) 198(10):4046–53. 10.4049/jimmunol.1601896 PMC5450662 28373583

[12] Klein SL Pekosz APC Anker M. P. O. Sex, Gender and Influenza. Geneva: World Health Organization (2010). p. 1–58.

[13] Serfling RE Sherman IL Houseworth WJ. Excess Pneumonia-Influenza Mortality by Age and Sex in Three Major Influenza A2 Epidemics, United States, 1957-58, 1960 and 1963. Am J Epidemiol (1967) 86(2):433–41. 10.1093/oxfordjournals.aje.a120753 6058395

[14] Ruggieri A Barbati C Malorni W. Cellular and Molecular Mechanisms Involved in Hepatocellular Carcinoma Gender Disparity. Int J Cancer (2010) 127(3):499–504. 10.1002/ijc.25298 20201099

[15] Tsay PK Tai DI Chen YM Yu CP Wan SY Shen YJ. Impact of Gender, Viral Transmission and Aging in the Prevalence of Hepatitis B Surface Antigen. Chang Gung Med J (2009) 32(2):155–64. 19403005

[16] Breidbart S Burk RD Saenger P. Hormonal Regulation of Hepatitis B Virus Gene Expression: Influence of Androgen Receptor. Pediatr Res (1993) 34(3):300–2. 10.1203/00006450-199309000-00012 8134171

[17] Ruggieri A Gagliardi MC Anticoli S. Sex-Dependent Outcome of Hepatitis B and C Viruses Infections: Synergy of Sex Hormones and Immune Responses? Front Immunol (2018) 9:2302. 10.3389/fimmu.2018.02302 30349537 PMC6186821

[18] Scarselli E Ansuini H Cerino R Roccasecca RM Acali S Filocamo G. The Human Scavenger Receptor Class B Type I is a Novel Candidate Receptor for the Hepatitis C Virus. EMBO J (2002) 21(19):5017–25. 10.1093/emboj/cdf529 PMC129051 12356718

[19] Hayashida K Shoji I Deng L Jiang DP Ide YH Hotta H. 17β-Estradiol Inhibits the Production of Infectious Particles of Hepatitis C Virus. Microbiol Immunol (2010) 54(11):684–90. 10.1111/j.1348-0421.2010.00268.x 21044142

[20] Murakami Y Fukasawa M Kaneko Y Suzuki T Wakita T Fukazawa H. Selective Estrogen Receptor Modulators Inhibit Hepatitis C Virus Infection At Multiple Steps of the Virus Life Cycle. Microbes Infect (2013) 15(1):45–55. 10.1016/j.micinf.2012.10.003 23103222

[21] Feng Y Ling Y Bai T Xie Y Huang J Li J. Covid-19 With Different Severity: A Multi-Center Study of Clinical Features. Am J Respir Crit Care Med (2020) 201:1380–8. 10.1164/rccm.202002-0445OC PMC7258639 32275452

[22] Garg S Kim L Whitaker M O’Halloran A Cummings C Holstein R. Hospitalization Rates and Characteristics of Patients Hospitalized With Laboratory-Confirmed Coronavirus Disease 2019 - COVID-NET, 14 States, March 1-30, 2020. MMWR Morb Mortal Wkly Rep (2020) 69(15):458–64. 10.15585/mmwr.mm6915e3 PMC7755063 32298251

[23] Abad-Díez JM Calderón-Larrañaga A Poncel-Falcó A Poblador-Plou B Calderón-Meza JM Sicras-Mainar A. Age and Gender Differences in the Prevalence and Patterns of Multimorbidity in the Older Population. BMC Geriatr (2014) 14:75. 10.1186/1471-2318-14-75 24934411 PMC4070347

[24] Gale EA Gillespie KM. Diabetes and Gender. Diabetologia (2001) 44(1):3–15. 10.1007/s001250051573 11206408

[25] Han MK Postma D Mannino DM Giardino ND Buist S Curtis JL. Gender and Chronic Obstructive Pulmonary Disease: Why it Matters. Am J Respir Crit Care Med (2007) 176(12):1179–84. 10.1164/rccm.200704-553CC PMC2720110 17673696

[26] Podcasy JL Epperson CN. Considering Sex and Gender in Alzheimer Disease and Other Dementias. Dialog Clin Neurosci (2016) 18(4):437–46. 10.31887/DCNS.2016.18.4/cepperson PMC5286729 28179815

[27] Li J Chen X McClusky R Ruiz-Sundstrom M Itoh Y Umar S. The Number of X Chromosomes Influences Protection From Cardiac Ischaemia/Reperfusion Injury in Mice: One X is Better Than Two. Cardiovasc Res (2014) 102(3):375–84. 10.1093/cvr/cvu064 PMC4030514 24654234

[28] Ragab D Salah Eldin H Taeimah M Khattab R Salem R. The COVID-19 Cytokine Storm; What We Know So Far. Front Immunol (2020) 11:1446. 10.3389/fimmu.2020.01446 32612617 PMC7308649

[29] Farooqi S Dickhout JG. Major Comorbid Disease Processes Associated With Increased Incidence of Acute Kidney Injury. World J Nephrol (2016) 5(2):139–46. 10.5527/wjn.v5.i2.139 PMC4777784 26981437

[30] Raza A Estepa A Chan V Jafar MS. Acute Renal Failure in Critically Ill COVID-19 Patients With a Focus on the Role of Renal Replacement Therapy: A Review of What We Know So Far. Cureus (2020) 12(6):e8429. 10.7759/cureus.8429 32642345 PMC7336623

[31] Coggins CH Breyer Lewis J Caggiula AW Castaldo LS Klahr S Wang SR. Differences Between Women and Men With Chronic Renal Disease. Nephrol Dial Transplant (1998) 13(6):1430–7. 10.1093/ndt/13.6.1430 9641172

[32] Guan WJ Ni ZY Hu Y Liang WH Ou CQ He JX. Clinical Characteristics of Coronavirus Disease 2019 in China. N Engl J Med (2020) 58:711–2. 10.1056/NEJMoa2002032 PMC7092819 32109013

[33] Huang C Wang Y Li X Ren L Zhao J Hu Y. Clinical Features of Patients Infected With 2019 Novel Coronavirus in Wuhan, China. Lancet (2020) 395(10223):497–506. 10.1016/S0140-6736(20)30183-5 31986264 PMC7159299

[34] Wehbe Z Hammoud S Soudani N Zaraket H El-Yazbi A Eid AH. Molecular Insights Into SARS Cov-2 Interaction With Cardiovascular Disease: Role of RAAS and MAPK Signaling. Front Pharmacol (2020) 11:836. 10.3389/fphar.2020.00836 32581799 PMC7283382

[35] Zheng YY Ma YT Zhang JY Xie X. Covid-19 and the Cardiovascular System. Nat Rev Cardiol (2020) 17:259–60. 10.1038/s41569-020-0360-5 PMC7095524 32139904

[36] Woodward M. Cardiovascular Disease and the Female Disadvantage. Int J Environ Res Public Health (2019) 16(7):1165. 10.3390/ijerph16071165 PMC6479531 30939754

[37] Möller-Leimkühler AM. Gender Differences in Cardiovascular Disease and Comorbid Depression. Dialog Clin Neurosci (2007) 9(1):71. 10.31887/DCNS.2007.9.1/ammoeller PMC3181845 17506227

[38] Pullen AB Kain V Serhan CN Halade GV. Molecular and Cellular Differences in Cardiac Repair of Male and Female Mice. J Am Heart Assoc (2020) 9(8):e015672. 10.1161/JAHA.119.015672 32295449 PMC7428548

[39] Bansal M. Cardiovascular Disease and COVID-19. Diabetes Metab Syndr (2020) 14(3):247–50. 10.1016/j.dsx.2020.03.013 PMC7102662 32247212

[40] Chen Y Guo Y Pan Y Zhao ZJ. Structure Analysis of the Receptor Binding of 2019-nCoV. Biochem Biophys Res Commun (2020) 525:135–40. 10.1016/j.bbrc.2020.02.071 PMC7092824 32081428

[41] Fu Y Cheng Y Wu Y. Understanding SARS-CoV-2-Mediated Inflammatory Responses: From Mechanisms to Potential Therapeutic Tools. Virol Sin (2020) 35:266–71. 10.1007/s12250-020-00207-4 PMC7090474 32125642

[42] Kuba K Imai Y Rao S Gao H Guo F Guan B. A Crucial Role of Angiotensin Converting Enzyme 2 (ACE2) in SARS Coronavirus-Induced Lung Injury. Nat Med (2005) 11(8):875–9. 10.1038/nm1267 PMC7095783 16007097

[43] Imai Y Kuba K Rao S Huan Y Guo F Guan B. Angiotensin-Converting Enzyme 2 Protects From Severe Acute Lung Failure. Nature (2005) 436(7047):112–6. 10.1038/nature03712 PMC7094998 16001071

[44] Brojakowska A Narula J Shimony R Bander J. Clinical Implications of SARS-CoV-2 Interaction With Renin Angiotensin System: Jacc Review Topic of the Week. J Am Coll Cardiol (2020) 75(24):3085–95. 10.1016/j.jacc.2020.04.028 PMC7161517 32305401

[45] Gemmati D Bramanti B Serino ML Secchiero P Zauli G Tisato V. Covid-19 and Individual Genetic Susceptibility/Receptivity: Role of ACE1/ACE2 Genes, Immunity, Inflammation and Coagulation. Might the Double X-Chromosome in Females be Protective Against SARS-CoV-2 Compared to the Single X-Chromosome in Males? Int J Mol Sci (2020) 21(10):3474. 10.3390/ijms21103474 PMC7278991 32423094

[46] Asselta R Paraboschi EM Mantovani A Duga S. ACE2 and TMPRSS2 variants and expression as candidates to sex and country differences in COVID-19 severity in Italy. Aging (Albany NY) (2020) 12(11):10087–98. 10.18632/aging.103415 PMC7346072 32501810

[47] Xie X Xudong X Chen J Junzhu C Wang X Xingxiang W. Age- and Gender-Related Difference of ACE2 Expression in Rat Lung. Life Sci (2006) 78(19):2166–71. 10.1016/j.lfs.2005.09.038 PMC7094566 16303146

[48] Cristiani L Mancino E Matera L Nenna R Pierangeli A Scagnolari C. Will Children Reveal Their Secret? The Coronavirus Dilemma. Eur Respir J (2020) 55(4). 10.1183/13993003.00749-2020 PMC7113798 32241833

[49] Coutard B Valle C de Lamballerie X Canard B Seidah NG Decroly E. The Spike Glycoprotein of the New Coronavirus 2019-nCoV Contains a Furin-Like Cleavage Site Absent in CoV of the Same Clade. Antiviral Res (2020) 176:104742. 10.1016/j.antiviral.2020.104742 32057769 PMC7114094

[50] Johnson BA Xie X Kalveram B Lokugamage KG Muruato A Zou J. Furin Cleavage Site Is Key to SARS-CoV-2 Pathogenesis. (2020). 10.1101/2020.08.26.268854 PMC8175039 33494095

[51] Cantuti-Castelvetri L Ojha R Pedro LD Djannatian M Franz J Kuivanen S. Neuropilin-1 Facilitates SARS-CoV-2 Cell Entry and Infectivity. Science (2020) 370(6518):856–60. 10.1126/science.abd2985 PMC7857391 33082293

[52] Lu J Cheng Y Zhang G Tang Y Dong Z McElwee KJ. Increased Expression of Neuropilin 1 in Melanoma Progression and its Prognostic Significance in Patients With Melanoma. Mol Med Rep (2015) 12(2):2668–76. 10.3892/mmr.2015.3752 PMC4464456 25954957

[53] Kang JY Gil M Kim KE. Neuropilin1 Expression Acts as a Prognostic Marker in Stomach Adenocarcinoma by Predicting the Infiltration of Treg Cells and M2 Macrophages. J Clin Med (2020) 9(5):1430. 10.3390/jcm9051430 PMC7290937 32408477

[54] Cao H Li Y Huang L Bai B Xu Z. Clinicopathological Significance of Neuropilin 1 Expression in Gastric Cancer: A Meta-Analysis. Dis Markers (2020) 2020:4763492. 10.1155/2020/4763492 33014187 PMC7520665

[55] Alattar M Omo A Elsharawy M Li J. Neuropilin-1 Expression in Squamous Cell Carcinoma of the Oesophagus. Eur J Cardiothorac Surg (2014) 45(3):514–20. 10.1093/ejcts/ezt380 23956271

[56] Wang K Chen W Zhang Z Deng Y Lian JQ Du P. CD147-Spike Protein is a Novel Route for SARS-CoV-2 Infection to Host Cells. Signal Transd Target Ther (2020) 5(1):283. 10.1038/s41392-020-00426-x PMC7714896 33277466

[57] Masre SF Jufri NF Ibrahim FW Abdul Raub SH. Classical and Alternative Receptors for SARS-CoV-2 Therapeutic Strategy. Rev Med Virol (2020) 27:e2207. 10.1002/rmv.2207 PMC7883063 33368788

[58] Radzikowska U Ding M Tan G Zhakparov D Peng Y Wawrzyniak P. Distribution of ACE2, Cd147, CD26, and Other SARS-CoV-2 Associated Molecules in Tissues and Immune Cells in Health and in Asthma, COPD, Obesity, Hypertension, and COVID-19 Risk Factors. Allergy (2020) 75(11):2829–45. 10.1111/all.14429 PMC7300910 32496587

[59] Zheng HC Gong BC. CD147 Expression was Positively Linked to Aggressiveness and Worse Prognosis of Gastric Cancer: A Meta and Bioinformatics Analysis. Oncotarget (2017) 8(52):90358–70. 10.18632/oncotarget.20089 PMC5685756 29163835

[60] Almeida LF Tofteng SS Madsen K Jensen BL. Role of the Renin-Angiotensin System in Kidney Development and Programming of Adult Blood Pressure. Clin Sci (Lond) (2020) 134(6):641–56. 10.1042/CS20190765 32219345

[61] Sparks MA Crowley SD Gurley SB Mirotsou M Coffman TM. Classical Renin-Angiotensin System in Kidney Physiology. Compr Physiol (2014) 4(3):1201–28. 10.1002/cphy.c130040 PMC4137912 24944035

[62] Hamming I Timens W Bulthuis ML Lely AT Navis G van Goor H. Tissue Distribution of ACE2 Protein, the Functional Receptor for SARS Coronavirus. A First Step Understanding SARS Pathogen J Pathol (2004) 203(2):631–7. 10.1002/path.1570 PMC7167720 15141377

[63] Simões e Silva AC Silveira KD Ferreira AJ Teixeira MM. ACE2, Angiotensin-(1-7) and Mas Receptor Axis in Inflammation and Fibrosis. Br J Pharmacol (2013) 169(3):477–92. 10.1111/bph.12159 PMC3682698 23488800

[64] Paz Ocaranza M Riquelme JA García L Jalil JE Chiong M Santos RAS. Counter-Regulatory Renin-Angiotensin System in Cardiovascular Disease. Nat Rev Cardiol (2020) 17(2):116–29. 10.1038/s41569-019-0244-8 PMC7097090 31427727

[65] Rocha NP Simoes E Silva AC Prestes TRR Feracin V Machado CA Ferreira RN. RAS in the Central Nervous System: Potential Role in Neuropsychiatric Disorders. Curr Med Chem (2018) 25(28):3333–52. 10.2174/0929867325666180226102358 29484978

[66] Kangussu LM Marzano LAS Souza CF Dantas CC Miranda AS Simões E Silva AC. The Renin-Angiotensin System and the Cerebrovascular Diseases: Experimental and Clinical Evidence. Protein Pept Lett (2019) 27:463–75. 10.2174/0929866527666191218091823 31849284

[67] Tikellis C Thomas MC. Angiotensin-Converting Enzyme 2 (Ace2) Is a Key Modulator of the Renin Angiotensin System in Health and Disease. Int J Pept (2012) 2012:256294. 10.1155/2012/256294 22536270 PMC3321295

[68] Cohall DH Scantlebury-Manning T James S Hall K Ferrario CM. Renin-Angiotensin-Aldosterone System Gender Differences in an Afro-Caribbean Population. J Renin Angiotensin Aldosterone Syst (2015) 16(3):539–46. 10.1177/1470320314523659 PMC4282614 24532825

[69] Sullivan JC Rodriguez-Miguelez P Zimmerman MA Harris RA. Differences in Angiotensin (1-7) Between Men and Women. Am J Physiol Heart Circ Physiol (2015) 308(9):H1171–6. 10.1152/ajpheart.00897.2014 PMC4551129 25659489

[70] Imanpour H Rezaee H Nouri-Vaskeh M. Angiotensin 1-7: A Novel Strategy in COVID-19 Treatment. Adv Pharm Bull (2020) 10(4):488–9. 10.34172/apb.2020.068 PMC7539308 33062600

[71] Muslin AJ. MAPK Signalling in Cardiovascular Health and Disease: Molecular Mechanisms and Therapeutic Targets. Clin Sci (Lond) (2008) 115(7):203–18. 10.1042/CS20070430 PMC2707780 18752467

[72] Bueno OF De Windt LJ Tymitz KM Witt SA Kimball TR Klevitsky R. The MEK1-ERK1/2 Signaling Pathway Promotes Compensated Cardiac Hypertrophy in Transgenic Mice. EMBO J (2000) 19(23):6341–50. 10.1093/emboj/19.23.6341 PMC305855 11101507

[73] Haq S Choukroun G Lim H Tymitz KM del Monte F Gwathmey J. Differential Activation of Signal Transduction Pathways in Human Hearts With Hypertrophy Versus Advanced Heart Failure. Circulation (2001) 103(5):670–7. 10.1161/01.cir.103.5.670 11156878

[74] Esposito G Prasad SV Rapacciuolo A Mao L Koch WJ Rockman HA. Cardiac Overexpression of a G(q) Inhibitor Blocks Induction of Extracellular Signal-Regulated Kinase and C-Jun NH(2)-terminal Kinase Activity in In Vivo Pressure Overload. Circulation (2001) 103(10):1453–8. 10.1161/01.cir.103.10.1453 11245652

[75] Gao JH Wang CH Tong H Wen SL Huang ZY Tang CW. Targeting Inhibition of Extracellular Signal-Regulated Kinase Kinase Pathway With AZD6244 (Arry-142886) Suppresses Growth and Angiogenesis of Gastric Cancer. Sci Rep (2015) 5:16382. 10.1038/srep16382 26567773 PMC4644956

[76] Mohammad G Mairaj Siddiquei M Imtiaz Nawaz M Abu El-Asrar AM. The ERK1/2 Inhibitor U0126 Attenuates Diabetes-Induced Upregulation of MMP-9 and Biomarkers of Inflammation in the Retina. J Diabetes Res (2013) 2013:658548. 10.1155/2013/658548 23671886 PMC3647581

[77] Cao Z Tong X Xia W Chen L Zhang X Yu B. CXCR7/P-ERK-Signaling Is a Novel Target for Therapeutic Vasculogenesis in Patients With Coronary Artery Disease. PloS One (2016) 11(9):e0161255. 10.1371/journal.pone.0161255 27612090 PMC5017667

[78] Cai Y Liu Y Zhang X. Suppression of Coronavirus Replication by Inhibition of the MEK Signaling Pathway. J Virol (2007) 81(2):446–56. 10.1128/JVI.01705-06 PMC1797436 17079328

[79] Icard P Lincet H Wu Z Coquerel A Forgez P Alifano M. The Key Role of Warburg Effect in SARS-CoV-2 Replication and Associated Inflammatory Response. Biochimie (2021) 180:169–77. 10.1016/j.biochi.2020.11.010 PMC7659517 33189832

[80] Varshney B Lal SK. Sars-CoV Accessory Protein 3b Induces AP-1 Transcriptional Activity Through Activation of JNK and ERK Pathways. Biochemistry (2011) 50(24):5419–25. 10.1021/bi200303r 21561061

[81] Ehrlichman LK Ford JW Roelofs KJ Tedeschi-Filho W Futchko JS Ramacciotti E. Gender-Dependent Differential Phosphorylation in the ERK Signaling Pathway is Associated With Increased MMP2 Activity in Rat Aortic Smooth Muscle Cells. J Surg Res (2010) 160(1):18–24. 10.1016/j.jss.2009.03.095 19592018 PMC2849897

[82] Pergola C Dodt G Rossi A Neunhoeffer E Lawrenz B Northoff H. ERK-Mediated Regulation of Leukotriene Biosynthesis by Androgens: A Molecular Basis for Gender Differences in Inflammation and Asthma. Proc Natl Acad Sci USA (2008) 105(50):19881–6. 10.1073/pnas.0809120105 PMC2597692 19064924

[83] Thiel V Weber F. Interferon and Cytokine Responses to SARS-coronavirus Infection. Cytokine Growth Factor Rev (2008) 19(2):121–32. 10.1016/j.cytogfr.2008.01.001 PMC7108449 18321765

[84] Dong G Fan H Yang Y Zhao G You M Wang T. 17β-Estradiol Enhances the Activation of IFN-α Signaling in B Cells by Down-Regulating the Expression of let-7e-5p, miR-98-5p and miR-145a-5p That Target Ikkϵ. Biochim Biophys Acta (2015) 1852(8):1585–98. 10.1016/j.bbadis.2015.04.019 25912736

[85] Kovats S. Estrogen Receptors Regulate Innate Immune Cells and Signaling Pathways. Cell Immunol (2015) 294(2):63–9. 10.1016/j.cellimm.2015.01.018 PMC4380804 25682174

[86] Jia HP Look DC Shi L Hickey M Pewe L Netland J. ACE2 Receptor Expression and Severe Acute Respiratory Syndrome Coronavirus Infection Depend on Differentiation of Human Airway Epithelia. J Virol (2005) 79(23):14614–21. 10.1128/JVI.79.23.14614-14621.2005 PMC1287568 16282461

[87] Hoffmann M Kleine-Weber H Schroeder S Krüger N Herrler T Erichsen S. SARS-Cov-2 Cell Entry Depends on ACE2 and TMPRSS2 and Is Blocked by a Clinically Proven Protease Inhibitor. Cell (2020) 181:271–80. 10.1016/j.cell.2020.02.052 PMC7102627 32142651

[88] Jia HP Look DC Tan P Shi L Hickey M Gakhar L. Ectodomain Shedding of Angiotensin Converting Enzyme 2 in Human Airway Epithelia. Am J Physiol Lung Cell Mol Physiol (2009) 297(1):L84–96. 10.1152/ajplung.00071.2009 PMC2711803 19411314

[89] Haga S Yamamoto N Nakai-Murakami C Osawa Y Tokunaga K Sata T. Modulation of TNF-alpha-converting Enzyme by the Spike Protein of SARS-CoV and ACE2 Induces TNF-alpha Production and Facilitates Viral Entry. Proc Natl Acad Sci USA (2008) 105(22):7809–14. 10.1073/pnas.0711241105 PMC2409424 18490652

[90] Menghini R Fiorentino L Casagrande V Lauro R Federici M. The Role of ADAM17 in Metabolic Inflammation. Atherosclerosis (2013) 228(1):12–7. 10.1016/j.atherosclerosis.2013.01.024 23384719

[91] Düsterhöft S Lokau J Garbers C. The Metalloprotease ADAM17 in Inflammation and Cancer. Pathol Res Pract (2019) 215(6):152410. 10.1016/j.prp.2019.04.002 30992230

[92] Li Y Yang C Ma G Cui L Gu X Chen Y. Analysis of ADAM17 Polymorphisms and Susceptibility to Sporadic Abdominal Aortic Aneurysm. Cell Physiol Biochem (2014) 33(5):1426–38. 10.1159/000358708 24853957

[93] Mollica V Rizzo A Massari F. The Pivotal Role of TMPRSS2 in Coronavirus Disease 2019 and Prostate Cancer. Future Oncol (2020) 16(27):2029–33. 10.2217/fon-2020-0571 PMC7359420 32658591

[94] Clinckemalie L Spans L Dubois V Laurent M Helsen C Joniau S. Androgen Regulation of the TMPRSS2 Gene and the Effect of a SNP in an Androgen Response Element. Mol Endocrinol (2013) 27(12):2028–40. 10.1210/me.2013-1098 PMC5426606 24109594

[95] Piprek RP Damulewicz M Tassan JP Kloc M Kubiak JZ. Transcriptome Profiling Reveals Male- and Female-Specific Gene Expression Pattern and Novel Gene Candidates for the Control of Sex Determination and Gonad Development in Xenopus Laevis. Dev Genes Evol (2019) 229(2-3):53–72. 10.1007/s00427-019-00630-y 30972573 PMC6500517

[96] Kermani NZ Song WJ Badi Y Versi A Guo Y Sun K. Sputum ACE2, TMPRSS2 and FURIN Gene Expression in Severe Neutrophilic Asthma. Respir Res (2021) 22(1):10. 10.1186/s12931-020-01605-8 33413387 PMC7788167

[97] Ghosh S Klein RS. Sex Drives Dimorphic Immune Responses to Viral Infections. J Immunol (2017) 198(5):1782–90. 10.4049/jimmunol.1601166 PMC5325721 28223406

[98] Newton AH Cardani A Braciale TJ. The Host Immune Response in Respiratory Virus Infection: Balancing Virus Clearance and Immunopathology. Semin Immunopathol (2016) 38(4):471–82. 10.1007/s00281-016-0558-0 PMC4896975 26965109

[99] Horvath CM. The Jak-STAT Pathway Stimulated by Interferon Alpha or Interferon Beta. Sci STKE (2004) 2004(260). 10.1126/stke.2602004tr10 15561979

[100] Yoo JK Kim TS Hufford MM Braciale TJ. Viral Infection of the Lung: Host Response and Sequelae. J Allergy Clin Immunol (2013) 132(6):1263–76. 10.1016/j.jaci.2013.06.006 PMC3844062 23915713

[101] Klein SL. Sex Influences Immune Responses to Viruses, and Efficacy of Prophylaxis and Treatments for Viral Diseases. Bioessays (2012) 34(12):1050–9. 10.1002/bies.201200099 PMC4120666 23012250

[102] Melgert BN Oriss TB Qi Z Dixon-McCarthy B Geerlings M Hylkema MN. Macrophages: Regulators of Sex Differences in Asthma? Am J Respir Cell Mol Biol (2010) 42(5):595–603. 10.1165/rcmb.2009-0016OC 19574533 PMC2874445

[103] Pisitkun P Deane JA Difilippantonio MJ Tarasenko T Satterthwaite AB Bolland S. Autoreactive B Cell Responses to RNA-related Antigens Due to TLR7 Gene Duplication. Science (2006) 312(5780):1669–72. 10.1126/science.1124978 16709748

[104] Berghöfer B Frommer T Haley G Fink L Bein G Hackstein H. TLR7 Ligands Induce Higher IFN-alpha Production in Females. J Immunol (2006) 177(4):2088–96. 10.4049/jimmunol.177.4.2088 16887967

[105] Fish EN. The X-files in Immunity: Sex-Based Differences Predispose Immune Responses. Nat Rev Immunol (2008) 8(9):737–44. 10.1038/nri2394 PMC7097214 18728636

[106] Klein SL Jedlicka A Fau - Pekosz A Pekosz A. The Xs and Y of Immune Responses to Viral Vaccines. Lancet Infect Dis (2010) 10:1474–4457. 10.1016/S1473-3099(10)70049-9 PMC6467501 20417416

[107] Weinstein Y Ran S Segal S. Sex-Associated Differences in the Regulation of Immune Responses Controlled by the MHC of the Mouse. J Immunol (1984) 132(2):656–61. 6228595

[108] Klein SL Flanagan KL. Sex Differences in Immune Responses. Nat Rev Immunol (2016) 16(10):626–38. 10.1038/nri.2016.90 27546235

[109] Butterworth M McClellan B Allansmith M. Influence of Sex in Immunoglobulin Levels. Nature (1967) 214(5094):1224–5. 10.1038/2141224a0 4169229

[110] Klein SL Marriott I Fish EN. Sex-Based Differences in Immune Function and Responses to Vaccination. Trans R Soc Trop Med Hyg (2015) 109(1):9–15. 10.1093/trstmh/tru167 25573105 PMC4447843

[111] Donnelly CA Bartley LM Ghani AC Le Fevre AM Kwong GP Cowling BJ. Gender Difference in HIV-1 RNA Viral Loads. HIV Med (2005) 6(3):170–8. 10.1111/j.1468-1293.2005.00285.x 15876283

[112] Hsieh AR Fann CS Yeh CT Lin HC Wan SY Chen YC. Effects of Sex and Generation on Hepatitis B Viral Load in Families With Hepatocellular Carcinoma. World J Gastroenterol (2017) 23(5):876–84. 10.3748/wjg.v23.i5.876 PMC5296204 28223732

[113] Hewagama A Patel D Yarlagadda S Strickland FM Richardson BC. Stronger Inflammatory/Cytotoxic T-cell Response in Women Identified by Microarray Analysis. Genes Immun (2009) 10(5):509–16. 10.1038/gene.2009.12 PMC2735332 19279650

[114] Tay MZ Poh CM Rénia L MacAry PA Ng LFP. The Trinity of COVID-19: Immunity, Inflammation and Intervention. Nat Rev Immunol (2020) 20:1–12. 10.1038/s41577-020-0311-8 32346093 PMC7187672

[115] di Mauro G Cristina S Concetta R Francesco R Annalisa C. Sars-Cov-2 Infection: Response of Human Immune System and Possible Implications for the Rapid Test and Treatment. Int Immunopharmacol (2020) 84:106519. 10.1016/j.intimp.2020.106519 32311668 PMC7161502

[116] Nikolich-Zugich J Knox KS Rios CT Natt B Bhattacharya D Fain MJ. Sars-CoV-2 and COVID-19 in Older Adults: What We may Expect Regarding Pathogenesis, Immune Responses, and Outcomes. GeroScience (2020) 42:1–10. 10.1007/s11357-020-00186-0 32274617 PMC7145538

[117] Shah VK Firmal P Alam A Ganguly D Chattopadhyay S. Overview of Immune Response During SARS-Cov-2 Infection: Lessons From the Past. Front Immunol (2020) 11:1949. 10.3389/fimmu.2020.01949 32849654 PMC7426442

[118] Zhang Y Xu J Jia R Yi C Gu W Liu P. Protective Humoral Immunity in SARS-CoV-2 Infected Pediatric Patients. Cell Mol Immunol (2020) 17(7):768–70. 10.1038/s41423-020-0438-3 PMC7203722 32382126

[119] Li G Chen X Xu A. Profile of Specific Antibodies to the SARS-associated Coronavirus. N Engl J Med (2003) 349(5):508–9. 10.1056/nejm200307313490520 12890855

[120] Wu HS Hsieh YC Su IJ Lin TH Chiu SC Hsu YF. Early Detection of Antibodies Against Various Structural Proteins of the SARS-associated Coronavirus in SARS Patients. J BioMed Sci (2004) 11(1):117–26. 10.1007/bf02256554 PMC7089234 14730215

[121] Roved J Westerdahl H Hasselquist D. Sex Differences in Immune Responses: Hormonal Effects, Antagonistic Selection, and Evolutionary Consequences. Horm Behav (2017) 88:95–105. 10.1016/j.yhbeh.2016.11.017 27956226

[122] Bhatia A Sekhon HK Kaur G. Sex Hormones and Immune Dimorphism. ScientificWorldJournal (2014) 2014:159150. 10.1155/2014/159150 25478584 PMC4251360

[123] Nakada D Oguro H Levi BP Ryan N Kitano A Saitoh Y. Oestrogen Increases Haematopoietic Stem-Cell Self-Renewal in Females and During Pregnancy. Nature (2014) 505(7484):555–8. 10.1038/nature12932 PMC4015622 24451543

[124] Zhou T Yang Z Chen Y Chen Y Huang Z You B. Estrogen Accelerates Cutaneous Wound Healing by Promoting Proliferation of Epidermal Keratinocytes Via Erk/Akt Signaling Pathway. Cell Physiol Biochem (2016) 38(3):959–68. 10.1159/000443048 26938432

[125] Xie H Hua C Sun L Zhao X Fan H Dou H. 17β-Estradiol Induces CD40 Expression in Dendritic Cells Via MAPK Signaling Pathways in a Minichromosome Maintenance Protein 6-Dependent Manner. Arthritis Rheum (2011) 63(8):2425–35. 10.1002/art.30420 21538324

[126] Xing D Miller A Novak L Rocha R Chen YF Oparil S. Estradiol and Progestins Differentially Modulate Leukocyte Infiltration After Vascular Injury. Circulation (2004) 109(2):234–41. 10.1161/01.Cir.0000105700.95607.49 14699005

[127] Robinson DP Hall OJ Nilles TL Bream JH Klein SL. 17β-estradiol protects females against influenza by recruiting neutrophils and increasing virus-specific cd8 t cell responses in the lungs. J Virol (2014) 88(9):4711–20. 10.1128/jvi.02081-13 PMC3993800 24522912

[128] Pan T Zhong L Wu S Cao Y Yang Q Cai Z. 17β-Oestradiol Enhances the Expansion and Activation of Myeloid-Derived Suppressor Cells Via Signal Transducer and Activator of Transcription (STAT)-3 Signalling in Human Pregnancy. Clin Exp Immunol (2016) 185(1):86–97. 10.1111/cei.12790 26969967 PMC4908292

[129] Peretz J Pekosz A Lane AP Klein SL. Estrogenic Compounds Reduce Influenza A Virus Replication in Primary Human Nasal Epithelial Cells Derived From Female, But Not Male, Donors. Am J Physiol Lung Cell Mol Physiol (2016) 310(5):L415–25. 10.1152/ajplung.00398.2015 PMC4773846 26684252

[130] Roberts CW Walker W Alexander J. Sex-Associated Hormones and Immunity to Protozoan Parasites. Clin Microbiol Rev (2001) 14(3):476–88. 10.1128/cmr.14.3.476-488.2001 PMC88985 11432809

[131] Maggio M Basaria S Ble A Lauretani F Bandinelli S Ceda GP. Correlation Between Testosterone and the Inflammatory Marker Soluble Interleukin-6 Receptor in Older Men. J Clin Endocrinol Metab (2006) 91(1):345–7. 10.1210/jc.2005-1097 16263825

[132] Malkin CJ Pugh PJ Jones RD Kapoor D Channer KS Jones TH. The Effect of Testosterone Replacement on Endogenous Inflammatory Cytokines and Lipid Profiles in Hypogonadal Men. J Clin Endocrinol Metab (2004) 89(7):3313–8. 10.1210/jc.2003-031069 15240608

[133] Bobjer J Katrinaki M Tsatsanis C Lundberg Giwercman Y Giwercman A. Negative Association Between Testosterone Concentration and Inflammatory Markers in Young Men: A Nested Cross-Sectional Study. PloS One (2013) 8(4):e61466. 10.1371/journal.pone.0061466 23637840 PMC3630214

[134] Gubbels Bupp MR Potluri T Fink AL Klein SL. The Confluence of Sex Hormones and Aging on Immunity. Front Immunol (2018) 9:1269. 10.3389/fimmu.2018.01269 29915601 PMC5994698

[135] Seillet C Laffont S Trémollières F Rouquié N Ribot C Arnal JF. The TLR-mediated Response of Plasmacytoid Dendritic Cells is Positively Regulated by Estradiol In Vivo Through Cell-Intrinsic Estrogen Receptor α Signaling. Blood (2012) 119(2):454–64. 10.1182/blood-2011-08-371831 22096248

[136] Lasso G Mayer SV Winkelmann ER Chu T Elliot O Patino-Galindo JA. A structure-informed atlas of human-virus interactions. Cell (2019) 178(6):1526–41.e1516. 10.1016/j.cell.2019.08.005 PMC6736651 31474372

[137] Montoya MC Krysan DJ. Repurposing Estrogen Receptor Antagonists for the Treatment of Infectious Disease. mBio (2018) 9(6). 10.1128/mBio.02272-18 PMC6299222 30563895

[138] Johansen LM Brannan JM Delos SE Shoemaker CJ Stossel A Lear C. Fda-approved selective estrogen receptor modulators inhibit ebola virus infection. Sci Transl Med (2013) 5190:190ra179. 10.1126/scitranslmed.3005471 PMC3955358 23785035

[139] Cham LB Friedrich SK Adomati T Bhat H Schiller M Bergerhausen M. Tamoxifen Protects From Vesicular Stomatitis Virus Infection. Pharmaceut (Basel) (2019) 12(4):142. 10.3390/ph12040142 PMC6958322 31547012

[140] Dyall J Coleman CM Hart BJ Venkataraman T Holbrook MR Kindrachuk J. Repurposing of clinically developed drugs for treatment of middle east respiratory syndrome coronavirus infection. Antimicrob Agents Chemother (2014) 58(8):4885–93. 10.1128/aac.03036-14 PMC4136000 24841273

[141] Ivanova MM Mazhawidza W Dougherty SM Minna JD Klinge CM. Activity and Intracellular Location of Estrogen Receptors Alpha and Beta in Human Bronchial Epithelial Cells. Mol Cell Endocrinol (2009) 305(1–2):12–21. 10.1016/j.mce.2009.01.021 19433257 PMC2767333

[142] Nhamoyebonde S Leslie A. Biological Differences Between the Sexes and Susceptibility to Tuberculosis. J Infect Dis (2014) 209 Suppl 3:S100–6. 10.1093/infdis/jiu147 24966189

[143] Oni T Gideon HP Bangani N Tsekela R Seldon R Wood K. Smoking, BCG and Employment and the Risk of Tuberculosis Infection in HIV-infected Persons in South Africa. PloS One (2012) 7(10):e47072. 10.1371/journal.pone.0047072 23056584 PMC3467259

[144] Anker M. Addressing Sex and Gender in Epidemic-Prone Infectious Diseases. Geneva: World Health Organ Rep (2007).

[145] Perumal R Naidoo K Padayatchi N. TB Epidemiology: Where are the Young Women? Know Your Tuberculosis Epidemic, Know Your Response. BMC Public Health (2018) 18(1):417. 10.1186/s12889-018-5362-4 29587706 PMC5872528

[146] Qiu F Liang CL Liu H Zeng YQ Hou S Huang S. Impacts of Cigarette Smoking on Immune Responsiveness: Up and Down or Upside Down? Oncotarget (2017) 8(1):268–84. 10.18632/oncotarget.13613 PMC5352117 27902485

[147] Yates TA Atkinson SH. Ironing Out Sex Differences in Tuberculosis Prevalence. Int J Tuberc Lung Dis (2017) 21(5):483–4. 10.5588/ijtld.17.0194 PMC5389340 28399960

[148] Romeo J Wärnberg J Marcos A. Drinking Pattern and Socio-Cultural Aspects on Immune Response: An Overview. Proc Nutr Soc (2010) 69(3):341–6. 10.1017/s0029665110001904 20598197

[149] Zeman MV Hiraki L Sellers EM. Gender Differences in Tobacco Smoking: Higher Relative Exposure to Smoke Than Nicotine in Women. J Womens Health Gend Based Med (2002) 11(2):147–53. 10.1089/152460902753645281 11975862

[150] Han J Chen X. A Meta-Analysis of Cigarette Smoking Prevalence Among Adolescents in China: 1981-2010. Int J Environ Res Public Health (2015) 12(5):4617–30. 10.3390/ijerph120504617 PMC4454929 25922989

[151] Ely M Hardy R Longford NT Wadsworth ME. Gender Differences in the Relationship Between Alcohol Consumption and Drink Problems are Largely Accounted for by Body Water. Alcohol Alcohol (1999) 34(6):894–902. 10.1093/alcalc/34.6.894 10659726

[152] Vardavas CI Nikitara K. Covid-19 and Smoking: A Systematic Review of the Evidence. Tob Induc Dis (2020) 18:20. 10.18332/tid/119324 32206052 PMC7083240

[153] Guo YR Cao QD Hong ZS Tan YY Chen SD Jin HJ. The Origin, Transmission and Clinical Therapies on Coronavirus Disease 2019 (COVID-19) Outbreak - an Update on the Status. Mil Med Res (2020) 7(1):11. 10.1186/s40779-020-00240-0 32169119 PMC7068984

[154] Fischinger S Boudreau CM Butler AL Streeck H Alter G. Sex Differences in Vaccine-Induced Humoral Immunity. Semin Immunopathol (2019) 41(2):239–49. 10.1007/s00281-018-0726-5 PMC6373179 30547182

[155] Harper A Flanagan KL. Effect of Sex on Vaccination Outcomes: Important But Frequently Overlooked. Curr Opin Pharmacol (2018) 41:122–7. 10.1016/j.coph.2018.05.009 29883854

[156] Fink AL Klein SL. Sex and Gender Impact Immune Responses to Vaccines Among the Elderly. Physiol (Bethesda) (2015) 30(6):408–16. 10.1152/physiol.00035.2015 PMC4630198 26525340

[157] Fink AL Klein SL. The Evolution of Greater Humoral Immunity in Females Than Males: Implications for Vaccine Efficacy. Curr Opin Physiol (2018) 6:16–20. 10.1016/j.cophys.2018.03.010 30320243 PMC6181235

[158] Krammer F. Sars-CoV-2 Vaccines in Development. Nature (2020) 586(7830):516–27. 10.1038/s41586-020-2798-3 32967006

[159] Brady E Nielsen MW Andersen JP Oertelt-Prigione S. Lack of Consideration of Sex and Gender in Clinical Trials for COVID-19. (2020). 10.1101/2020.09.13.20193680 PMC8260641 34230477

[160] Jackson LA Anderson EJ Rouphael NG Roberts PC Makhene M Coler RN. An Mrna Vaccine Against SARS-CoV-2 - Preliminary Report. N Engl J Med (2020) 383(20):1920–31. 10.1056/NEJMoa2022483 PMC7377258 32663912

[161] Folegatti PM Ewer KJ Aley PK Angus B Becker S Belij-Rammerstorfer S. Safety and Immunogenicity of the ChAdOx1 nCoV-19 Vaccine Against SARS-CoV-2: A Preliminary Report of a Phase 1/2, Single-Blind, Randomised Controlled Trial. Lancet (2020) 396(10249):467–78. 10.1016/s0140-6736(20)31604-4 PMC7445431 32702298

[162] Bar-Zeev N Moss WJ. Encouraging Results From Phase 1/2 COVID-19 Vaccine Trials. Lancet (2020) 396(10249):448–9. 10.1016/s0140-6736(20)31611-1 PMC7832572 32702300

